# Atlas of Clinical Medicine

**Published:** 1897-12

**Authors:** 


**Atlas of Clinical Medicine.** By Byrom Bramwell, M.D. Vol. III. Edinburgh: T. and A. Constable. 1896.

With this volume Dr. Bramwell brings his folio Atlas to an end. We may remind our readers that the book, besides being a storehouse of illustrations, is also a work on medicine, dealing, it is true, not with the whole of medicine, but still covering a wide range of subjects. These subjects are treated by the author in a masterly manner; he deals fully, in many cases exhaustively, with the matter under discussion, and the letterpress thus embodies the most recent researches and collates them with the older views, so that the book will be found a useful work of reference. It is a pleasure to read accounts of diseases which deal adequately with all sides of the subject, and without being diffuse do not sacrifice a full discussion to the exigencies of space.

Of the number and excellence of the illustrations we can only speak in terms of high praise. They bear on their face evidence of the care and trouble that has been taken to make them really valuable representations of diseased conditions. Clearly it has been a labour of love to the author, and he must be congratulated on the success that has attended his efforts. Many of the plates, *e.g.* those representing various types of insanity, are in themselves artistic; and mention must be made of the fidelity with which they figure the disease represented, and also of the excellent way in which they have been reproduced. This is only a deserved tribute to the plates, both of clinical and microscopical appearances. We know of no other work on medicine which is to be compared with it in this respect. On this account alone the book should have a wide circulation amongst the medical profession, and its accurate and life-like delineations of disease, in many instances of diseases but seldom met with, should enable its possessors practising in districts remote from the great medical centres to make the diagnosis in an obscure case which otherwise they might fail altogether to recognise.

To turn to the present volume, the chief subjects of which it treats are: syphilis, muscular atrophy, the progressive muscular dystrophies, cyanosis, the thyroid treatment of skin diseases, congenital heart disease, the various forms of anæmia, and a very remarkable, if not unique, case of calcareous degeneration of the heart and arteries. These diseases receive full discussion in the letterpress, and are copiously and beautifully illustrated. We may, perhaps, select the article on the progressive muscular dystrophies as particularly good in the account given of the symptomatology and pathology of these rare maladies, and in the way in which both the former and the latter are set forth in a fine series of plates. An excellent and well-considered summary of our present knowledge of the muscular dystrophies is made more valuable by a number of illustrative cases. This is in fact the method which the author has followed in most of his chapters, which also contain sections on the differential diagnosis and treatment, and a useful note of the points to be investigated in future clinical and pathological examinations. We ought not to pass over without special mention the pictures illustrative of the thyroid treatment of skin affections, and the beautifully coloured portraits of patients suffering from morbus cœruleus, mitral disease, alopecia, and forms of dementia.

Dr. Bramwell has earned our gratitude by bringing out this work, which must obviously have entailed great labour and expense, and we hope that his enterprise will meet with an adequate reward.

